# Measures to Predict The Individual Variability of Corticospinal Responses Following Transcranial Direct Current Stimulation

**DOI:** 10.3389/fnhum.2016.00487

**Published:** 2016-10-06

**Authors:** Nathan D. Nuzum, Ashlee M. Hendy, Aaron P. Russell, Wei-Peng Teo

**Affiliations:** ^1^School of Exercise and Nutrition Sciences, Deakin UniversityGeelong, VIC, Australia; ^2^Institute for Physical Activity and Nutrition (IPAN), Deakin UniversityGeelong, VIC, Australia

**Keywords:** anodal tDCS, transcranial magnetic stimulation, latency, I-waves, short intracortical facilitation

## Abstract

Individual responses to transcranial direct current stimulation (tDCS) are varied and therefore potentially limit its application. There is evidence that this variability is related to the contributions of Indirect waves (I-waves) recruited in the cortex. The latency of motor-evoked potentials (MEPs) can be measured through transcranial magnetic stimulation (TMS), allowing an individual’s responsiveness to tDCS to be determined. However, this single-pulse method requires several different orientations of the TMS coil, potentially affecting its reliability. Instead, we propose a paired-pulse TMS paradigm targeting I-waves as an alternative method. This method uses one orientation that reduces inter- and intra-trial variability. It was hypothesized that the paired-pulse method would correlate more highly to tDCS responses than the single-pulse method. In a randomized, double blinded, cross-over design, 30 healthy participants completed two sessions, receiving 20 min of either anodal (2 mA) or sham tDCS. TMS was used to quantify Short interval intracortical facilitation (SICF) at Inter stimulus intervals (ISIs) of 1.5, 3.5 and 4.5 ms. Latency was determined in the posterior-anterior (PA), anterior-posterior (AP) and latero-medial (LM) coil orientations. The relationship between latency, SICF measures and the change in suprathreshold MEP amplitude size following tDCS were determined with Pearson’s correlations. TMS measures, SICI and SICF were also used to determine responses to Anodal-tDCS (a-tDCS). Neither of the latency differences nor the SICF measures correlated to the change in MEP amplitude from pre-post tDCS (all *P* > 0.05). Overall, there was no significant response to tDCS in this cohort. This study highlights the need for testing the effects of various tDCS protocols on the different I-waves. Further research into SICF and whether it is a viable measure of I-wave facilitation is warranted.

## Introduction

Transcranial direct current stimulation (tDCS) is a non-invasive brain stimulation technique that can modulate corticospinal excitability in a polarity-dependent manner. Specifically, the application of anodal tDCS (a-tDCS) over the primary motor cortex (M1) can induce a facilitatory effect in motor-evoked potential (MEP) amplitude elicited by transcranial magnetic stimulation (TMS), while cathodal tDCS suppresses it. While the facilitatory and inhibitory effects on corticospinal excitability may hold therapeutic potential in clinical applications, a major limitation of tDCS lies in its inherent individual variability in its response.

The reasons behind the individual variability in responses to tDCS are not fully understood and it is likely to be attributed to different factors (i.e., skull thickness, time-of-day, tDCS protocols and/or neurotrophic factor polymorphisms). However, several studies to date have suggested that the dynamics of trans-synaptic inter-neuronal networks, particularly those of early and later indirect waves (I-waves) may, in part, explain the variability in tDCS responses. In particular, Wiethoff et al. (2014) suggested that people who showed a facilitatory response to a-tDCS are more likely to recruit early I-waves or direct waves (D-waves) compared to tDCS non-responders or those that do not respond in a “canonical” manner. Later studies have further demonstrated the relationship between early I-wave recruitment and a-tDCS response (McCambridge et al., 2015; Davidson et al., 2016), with McCambridge et al. (2015) finding the relationship only existing in the distal muscles of the upper limb (i.e., extensor carpi radialis (ECR)), not those of the proximal upper limb (i.e., biceps brachii). These studies used MEP latency differences, between different coil orientations, as a surrogate measure of I-wave recruitment (Wiethoff et al., 2014; McCambridge et al., 2015; Davidson et al., 2016).While it is unclear as to why such a relationship exists between early I-wave recruitment and a-tDCS response, one reason could be that a-tDCS depolarizes the cell bodies of pyramidal neurons for which early I-wave inputs are targeted (Wiethoff et al., 2014). Studies of patients implanted with high cervical epidural electrodes for pain suggested a-tDCS preferentially modulates cortical circuits generating D- and early I-wave activity (Lang et al., 2011; Di Lazzaro et al., 2013). These seminal studies therefore suggest a seemingly close interaction between early I-wave recruitment and a-tDCS responses.

Apart from using MEP latency differences between coil orientations, paired-pulse TMS that targets I-wave periodicity is another method of investigating the dynamics of trans-synaptic inter-neuronal networks. First described by Ziemann et al. (1998), paired-pulse TMS, at suprathreshold intensities, delivered at 1.1–1.5 ms, 3.0–3.5 ms and 4.1–4.5 ms interstimulus intervals (ISIs) produces a clear short-interval intracortical facilitation (SICF) that is due to the facilitatory interactions between I-waves (Thickbroom et al., 2006; Cash et al., 2009). While our current understanding of I-waves and its interaction is limited, generation of early and later I-waves are likely to have different underlying mechanism (for review see Di Lazzaro et al., 2012) that contribute towards the modulation of a-tDCS response. For example, early I-waves are thought to originate from monosynaptic activation of P5 pyramidal tract neurons (PTN) via more superficial P2–3 PTNs, and are not influenced by GABAergic inhibitory influences (Di Lazzaro et al., 2012). Later I-waves may be generated by reactivation of P5 PTNs via excitatory reciprocal connections with P2–3 PTNs and interneurons that have approximately 1.5 ms transmission delay (Thomson et al., 2002). These monosynaptic connections are further influenced by GABAergic interneuron connections, thus having a greater inhibitory influence (Di Lazzaro et al., 2000). Given that I-waves are thought to result from trans-synaptic activation of corticospinal neurons via excitatory cortical interneurons (Ziemann and Rothwell, 2000; Rusu et al., 2014; Wiethoff et al., 2014; McCambridge et al., 2015), and that the effects of a-tDCS are associated with the modulation of excitatory and inhibitory interneurons (Medeiros et al., 2012), the interactions between I-waves may have implications for a-tDCS, whereby its efficacy may be influenced by GABAergic mechanisms and also the level of excitability of more superficial PTNs that tDCS may have a greater effect on.

The aim of this exploratory study was to investigate the relationship between I-wave dynamics (i.e., I-wave recruitment and facilitation) with variability in a-tDCS responses. Specifically, we compared SICF (pre-tDCS) elicited using paired-pulse TMS at I-wave periodicities, and I-wave recruitment by measuring MEP latency differences at different TMS coil orientations, to changes in corticospinal excitability following a-tDCS. We hypothesized that measures of SICF at I-wave periodicities will correlate with changes in corticospinal excitability following a-tDCS in a similar fashion to the coil orientation method as previously reported (Wiethoff et al., 2014; McCambridge et al., 2015).

## Materials and Methods

### Participants

Thirty healthy participants (19 males, 11 females, aged 18–34 years, height: 173.1 ± 1.5 cm, weight: 71.6 ± 2.3 kg) completed two testing sessions, each lasting approximately 1.5 h in a randomized, double-blinded cross-over study. All participants received either real or sham a-tDCS in a randomized order, separated by a minimum 48 h washout period (Nitsche et al., 2008). Both researcher and participant were blinded to the condition of stimulation during each session. Written informed consent was obtained from participants prior to testing and participants were also screened for TMS eligibility prior to any testing. Any participants that were left-handed, as determined through the Edinburgh Handedness Questionnaire (Oldfield, 1971), or who had any contraindications to TMS or tDCS, such as implanted medical devices, or neurological conditions, were excluded from this study. Left-handed participants were excluded as right handed participants were selected for convenience sampling and to avoid any potential laterality effects. This study complied with the Declaration of Helsinki and was approved by the Deakin University Human Research Ethics Committee.

#### Study Design

**Figure 1:**
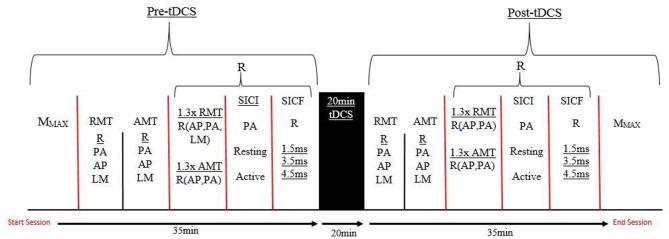
**A timeline of the testing protocol in both testing sessions.** R, Randomized order; AMT, Active motor threshold; RMT, Resting motor threshold; PA, Posterior-anterior; AP, Anterior-posterior; LM, Latero-medial; SICI, Short interval intracortical inhibiton; SICF, Short interval intracortical facilitation; tDCS, transcranial direct current stimulation; M_MAX_, Maximal muscle response.

Prior to the experiment, all participants were seated comfortably on a chair with their right arm relaxed and their elbow placed at a 90° angle on the arm rest. The optimal location of the ECR muscle was identified via palpation of the radial epicondyle and the styloid process of the ulnar and skin was prepared before attaching the surface electromyography (sEMG) electrodes. The researcher collected the maximal compound wave (M-wave) measure, before determining resting motor threshold (RMT) and active motor threshold (AMT) for the participant. Measurement of 130% of RMT and AMT was then collected in the posterior-anterior (PA) and anterior-posterior (AP) orientations, along with SICI in resting and active conditions and SICF at 1.5, 3.5 and 4.5 ms in a resting state. These measures were taken in a randomized order. Twenty minutes of tDCS (sham or a-tDCS) was applied before repeating the same TMS outcome measures. Figure 1 shows the timeline of experimental procedures for this study.

#### Surface Electromyography

sEMG activity was recorded from the ECR muscle of the right forearm. The location of the ECR muscle was determined through measuring from the humeral lateral epicondyle to the medial styloid process of the ulnar. The halfway point was determined, and recorded, while the participant was seated with their arm by their side at 90° of flexion at the elbow and with the hand pronated, in line with the forearm. Seven centimetres proximal to this point was palpated to determine where the bulk of the ECR muscle belly lay, according to the sEMG recording for non-invasive assessment of muscles (Stegeman and Hermens, 2007). This was marked with a surgical marker and recorded for correct placement in the following session. At the site of the ECR muscle, the participant’s skin was shaved of excess hair and scrubbed with an abrasive gel (Nuprep, Weaver and Co., Aurora, CO, USA) to remove any dead skin and cleaned with 70% isopropyl alcohol wipes. Positive and negative bipolar Ag-AgCl foam electrodes (Covidien, Quebec, QC, Canada) were placed onto this site in a muscle-belly tendon arrangement, with the positive electrode proximal to the negative electrode, with an inter-electrode distance of 2 cm according to SENIAM guidelines (Stegeman and Hermens, 2007). The electrodes were fastened with tape to prevent movement artifact, and a grounding electrode was connected around the wrist of the participant via a grounding velcro strap. All EMG signals were amplified (×1000) with band-pass filtering between 20 Hz and 1 kHz for 500 ms.

#### Maximal Compound Waves

To account for any peripheral changes at the muscle level, maximal compound waves were evoked from the ECR muscle using a peripheral nerve stimulator (DS7A, Digitimer, UK) applied to the radial nerve on the lateral aspect of the upper right arm, above the bend of the elbow. To ensure a maximal muscle response (M_MAX_) was obtained, the stimulation intensity was increased in 10% increments until no further increase was seen in the amplitude of the sEMG response (Goodwill et al., 2013). A further three stimulus was applied at this intensity to obtain an average M_MAX_ measure.

#### Transcranial Magnetic Stimulation Measures

Single- and paired-pulse TMS was delivered using a Bistim 200^2^ magnetic stimulator (Magstim Co, Dyfed, UK) with a figure-eight shaped coil, with an external loop diameter of 90 mm. The optimal site for the motor representation of the ECR muscle, or hotspot, was first identified through initial exploration using single-pulse TMS around the right hand representation of the left primary M1. The optimal hotspot was defined as the site that elicited the largest and most consistent MEPs during single-pulse TMS. Once the optimal hotspot was located, it was marked with a surgical marker, and recorded for consistency between pre- and post-testing, and to ensure repeatability between the sham and real tDCS sessions. 10 MEPs were collected at each stimulus intensity.

Single-pulse TMS was used to measure RMT and AMT. RMT was defined as the minimum percent of maximum stimulator output (%MSO) that will elicit an average MEP amplitude response between 50–100 μV in at least 5 out of 10 stimulations of the ECR muscle at rest (Rothwell et al., 1999). AMT was defined as the minimum %MSO required to elicit an average MEP amplitude between 200–300 μV in at least 5 out of 10 stimulations, when the ECR muscle is slightly activated at approximately 5 ± 2% of maximal sEMG (Rothwell et al., 1999). In the current study, this low-level of activation was achieved by maintaining the fingers and hand at 180° in line with the forearm, and was measured through the root mean squared EMG (rmsEMG; Hendy and Kidgell, 2014). Measurement at 130% of RMT and AMT was then undertaken in a PA and AP orientation, and at 130% of RMT in the latero-medial (LM) orientation. The MEP latency (time between TMS stimulus and onset of MEP) for each coil orientation, at 130% of RMT, was measured for each individual response, manually with a cursor. The participant’s average latency at each orientation (10 MEPs at each orientation) was then used to calculate the latency differences between those orientations and to determine the recruitment of I-waves (Hamada et al., 2013).

To measure short-interval intracortical inhibition (SICI), a conditioning stimulus (CS) at an intensity of 80% of RMT was followed with a testing stimulus (TS) at an intensity of 130% of RMT for the resting condition. During the active condition 80% of AMT and 130% of AMT was used for the CS and TS respectively. The CS and TS were separated by an ISI of 3 ms during both resting and active ECR conditions (Kujirai et al., 1993; Hummel et al., 2005; Hendy and Kidgell, 2014).

SICF was taken at resting and active ECR conditions, with both the CS and TS set to the RMT, and AMT for the resting and active conditions respectively. This was tested at ISIs of 1.5, 3.5 and 4.5 ms, which corresponds to the periodicity of the first, second and third I-waves respectively (Day et al., 1989; Tokimura et al., 1996; Ziemann et al., 1998; Thickbroom et al., 2006). An ISI interval of 3 ms was not used as this coincides with the ISI used to elicit SICI.

The amount of SICI was determined by calculating paired-pulse MEP as a percentage of the single-pulse MEP elicited at the same TS intensity. SICF was measured as a percentage difference of the paired-pulse SICF MEPs to the single-pulse MEPs (130% of RMT and 130% of AMT) and the difference from pre-post of 130% of RMT and AMT was used to determine tDCS responses.

#### Transcranial Direct Current Stimulation Protocol

tDCS (NeuroConn, Ilmenau, Germany) was applied to the participant for 20 min, at an intensity of 2 mA (0.08 mA/cm^2^; Iyer et al., 2005). Two 25 cm^2^ rubber electrode pads were attached in a bipolar electrode montage with the anode placed over the M1 corresponding with the right ECR muscle and the cathode placed over the participant’s right supraorbital area (Nitsche and Paulus, 2001). Both the anode and cathode were held in position using rubber straps for the duration of stimulation and an electrode conducting gel (Ten20 Conductive Gel, Weaver and Company, Aurora, CO, USA) was applied to the electrodes instead of saline solution to reduce electrical impedance.

For a-tDCS, the stimulus intensity was ramped up to 2 mA in the first 15 s and maintained for 20 min before ramping down in the last 15 s. Sham tDCS only incorporated the ramping up phase in the first 15 s to provide a similar sensation before ramping down (Hummel et al., 2005; Palm et al., 2013). This method of sham stimulation has been previously validated and is commonly used as a control for studies using tDCS (Gandiga et al., 2006). The tDCS device was programmed to deliver either real or sham stimulation, which the researcher activated with pre-programmed codes. The researcher conducting the testing sessions was only un-blinded to stimulation type once all data analysis was completed.

### Analytical Procedures and Statistical Analysis

#### Analytical Procedures

The primary outcome measures of this study were SICF, elicited by paired-pulse TMS I-wave periodicities, the latency differences between coil orientations, excitability (tDCS response) and inhibition (SICI) following tDCS. Corticospinal excitability is expressed as a percentage difference in MEP amplitude from pre to post-tDCS (Figure 9). MEP latency was determined visually, from the time when TMS is triggered to the onset of the MEP (McCambridge et al., 2015). The difference between the latencies of the coil orientations (AP-LM, PA-LM, AP-PA) were used to determine I-wave recruitment (Wiethoff et al., 2014; McCambridge et al., 2015). All outcome measures were analyzed using LabChart 8 software (ADinstruments, Bella Vista, NSW, Australia).

#### Statistical Analysis

The mean and standard error (SE) of participant’s RMT, AMT and %MSO values across both sessions were calculated. Kolmogorov-Smirnov and Shapiro-Wilk tests were used to determine normal distribution of data, and log transformations were applied where appropriate. A two-way repeated measures analysis of variance (ANOVA; CONDITION- sham vs. real; TIME- pre vs. post tDCS) was undertaken to test for significant effects of tDCS, SICF_ratio_ and SICI_ratio_ measures and Bonferroni *post hoc* analysis was conducted where appropriate and significance was found. Pearson product moment correlation was used to determine the relationship between latency differences (AP-PA, AP-LM and PA-LM), SICF_ratio_ (at inter-stimulus intervals (ISI) of 1.5, 3.5 and 4.5 ms), and tDCS response, in both active and resting muscle states. These analyses were done with MEPs normalized to M_MAX_. *T*-tests were performed between the ISI’s of the SICF measures post real-tDCS.

All statistical analysis was performed using SPSS version 22 (SPSS Inc., Chicago, IL, USA). An alpha level of *P* < 0.05 was set as the level of significance.

## Results

### Participants

#### M_MAX_

Participants M_MAX_ was recorded to ensure peripheral excitability remained constant throughout and between testing sessions. This was established through a range of 6–9.5 mA. There was no significant effect of TIME (*F*_(1,29)_ = 1.787, *P* = 0.192, *η*^2^ = 0.253), CONDITION (*F*_(1,29)_ = 0.113, *P* = 0.739, *η*^2^ = 0.062), nor was a significant TIME × CONDITION interaction present (*F*_(1,29)_ = 0.782, *P* = 0.384, *η*^2^ = 0.137).

#### Pre-Stim rmsEMG

Participants rmsEMG from the active conditions were compared to ensure consistent background muscle activity levels during testing, with no significant effect of TIME (*F*_(1,29)_ = 1.651, *P* = 0.209, *η*^2^ = 0.237), CONDITION (*F*_(1,29)_ = 0.055, *P* = 0.816, *η*^2^ = 0.056) or TIME × CONDITION (*F*_(1,29)_ = 0.317, *P* = 0.578, *η*^2^ = 0.085) interaction present.

#### Stimulator Output to Evoke AMT and RMT

The MEP amplitude obtained at RMT and AMT, as well as the percentage of maximum stimulator output (%MSO) required to evoke RMT and AMT for both sessions, are displayed in Table 1. No differences existed between MEPs or %MSO obtained either pre or post-tDCS in both sessions.

**Table 1:** **Transcranial magnetic stimulation (TMS) characteristics and responses to transcranial direct current stimulation (tDCS) (mean ± SE)**.

	**Real**	**Sham**
**RMT MEPs (mV)**		
Pre	0.094 ± 0.009	0.095 ± 0.007
Post	0.095 ± 0.014	0.103 ± 0.010
		
**M_max_ (mV)**		
Pre	7.9 ± 2.69	8.1 ± 2.46
Post	8.3 ± 2.47	8.2 ± 2.46
**AMT MEPs (mV)**		
Pre	0.533 ± 0.036	0.503 ± 0.033
Post	0.561 ± 0.048	0.520 ± 0.035
		
**RMT (%MSO)**		
Pre	45 ± 2	45 ± 2
Post	45 ± 2	44 ± 2
		
**AMT (%MSO)**		
Pre	38 ± 2	37 ± 2
Post	38 ± 2	37 ± 2
		
**Sensations**		
Pain	2 ± 0.4	2 ± 0.4
Tingling	3 ± 0.5	3 ± 0.5
Itching	2 ± 0.4	2 ± 0.4
Burning	4 ± 0.5	3 ± 0.5

#### Sensations of Real and Sham tDCS

Participant’s predictions as to which sessions were real and which were sham were calculated. Out of a possible 60 responses, participants correctly identified the tDCS condition 37 times (Correct guess 61% of the time). Participants were more likely to identify either session as real over sham, with 38 of the responses being real, and only 22 of the responses being sham. Responses to the sensation questionnaire (Supplementary Data Sheet 1) were compared between sessions and were not significantly different between the real and sham sessions (all *P* > 0.050).

### Corticospinal Responses Following Transcranial Direct Current Stimulation

#### Corticospinal Excitability in Active and Resting Conditions

**Figure 2:**
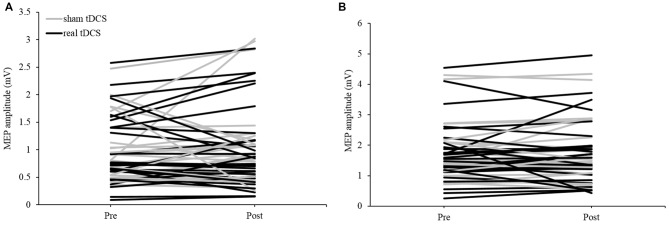
**Individual motor-evoked potential (MEP) responses following tDCS in (A) resting and (B) active extensor carpi radialis (ECR) conditions in both sham and real sessions**.

The group mean data under the active condition saw no significant main effect for TIME (*F*_(1,29)_ = 0.130, *P* = 0.721, *η*^2^ = 0.004), CONDITION (*F*_(1,29)_ = 0.029, *P* = 0.867, *η*^2^ = 0.001) nor was there a significant TIME × CONDITION interaction (*F*_(1,29)_ = 0.265, *P* = 0.610, *η*^2^ = 0.009). Mean group data showed the sham session (5.09% change) and the real session (7.56% change) both increased overall. Sixteen participants showed an increase in tDCS excitability in the real session and 15 displayed an increase in the sham session (Figure 2B). The responses for active MEP amplitude changes were less variable when compared to resting MEP amplitude changes (real tDCS from −43 to 127% change, sham tDCS from −35 to 97% change).

**Figure 3:**
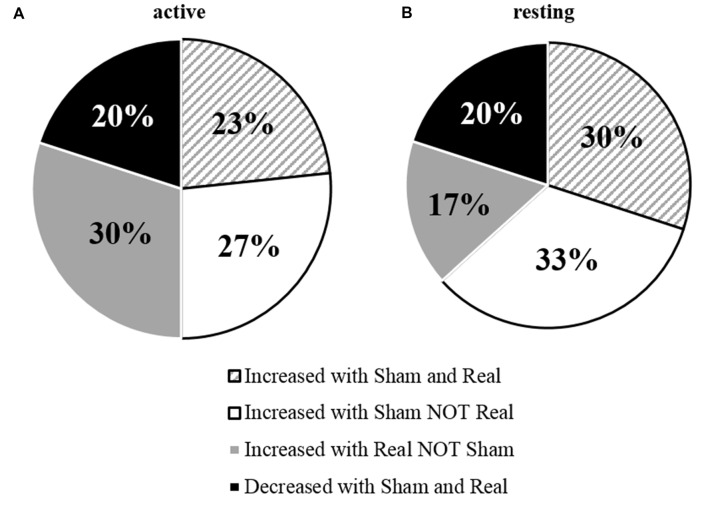
**Percentage of individual’s responses to tDCS measured under (A) active and (B) resting conditions**.

For the group mean data of tDCS responses in the resting condition, there was no significant main effect for TIME (*F*_(1,29)_ = 0.035, *P* = 0.852, *η*^2^ = 0.001), CONDITION (*F*_(1,29)_ = 0.017, *P* = 0.896, *η*^2^ = 0.017) nor was there a significant TIME × CONDITION interaction (*F*_(1,29)_ = 0.207, *P* = 0.652, *η*^2^ = 0.007). Across all participants there was a greater increase from pre-post in the sham session (24.93%) than in the real session (8.26%). Individually, 14 participants displayed an increase in excitability in the real session, while 19 displayed an increase during the sham session (Figure 2A). The amount of change between individuals was extremely variable under resting conditions (real tDCS from −65 to 135% change, sham tDCS from −85 to 271% change), with some participants responding negatively in both conditions, and others responding positively (Figure 3).

This data shows the overall response to tDCS was not significant. This appears to be due to high variability between individuals, as some participants showed positive changes to tDCS excitability from the real tDCS session.

#### Short Interval Intracortical Inhibition in Resting and Active Conditions

The magnitude of inhibition did not differ significantly between pre-post measures of SICI, or between the real and sham sessions. No significant main effects for TIME (*F*_(1,29)_ = 0.814, *P* = 0.374, *η*^2^ = 0.027), CONDITION (*F*_(1,29)_ = 0.026, *P* = 0.872, *η*^2^ = 0.001), or TIME × CONDITION interaction (*F*_(1,29)_ < 0.000, *P* = 0.999, *η*^2^ < 0.000) were seen for SICI under the active condition.

No significant main effects for TIME (*F*_(1,29)_ = 1.392, *P* = 0.248, *η*^2^ = 0.046), CONDITION (*F*_(1,29)_ = 1.078, *P* = 0.308, *η*^2^ = 0.036) or TIME × CONDITION interaction was seen during resting SICI (*F*_(1,29)_ = 0.168, *P* = 0.685, *η*^2^ = 0.006; Figure 8).

SICI, at baseline, was significant across all measures of SICI, including SICI at rest with real tDCS (*F*_(1,29)_ = 84.048, *P* < 0.001, *η*^2^ = 1.000) and sham tDCS (*F*_(1,29)_ = 72.215, *P* < 0.001, *η*^2^ = 1.000) and during the active condition with real tDCS (*F*_(1,29)_ = 176.562, *P* < 0.001, *η*^2^ = 1.000) and sham tDCS (*F*_(1,29)_ = 228.005, *P* < 0.001, *η*^2^ = 1.000).

#### Short Interval Intracortical Facilitation in Resting Conditions

**Figure 4:**
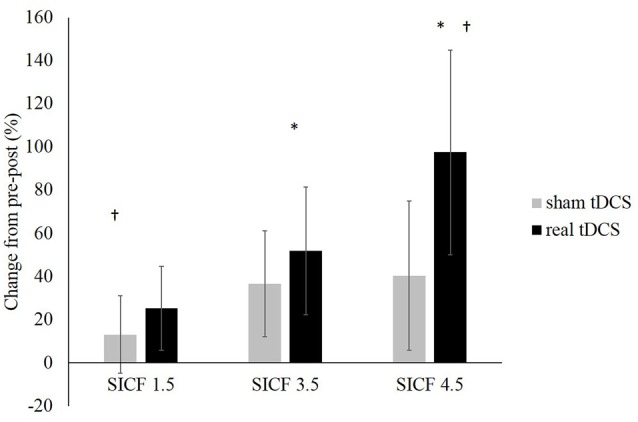
**Change in SICF MEP amplitudes (as a percentage of unconditioned MEPs) at ISIs of 1.5, 3.5 and 4.5 ms (all** *P*** > 0.05).** *Denotes significant difference between SICF 3.5 and 4.5. ^†^Denotes significant difference between SICF 1.5 and 4.5.

From pre-post tDCS there was no significant change, with no effect of the SICF protocol (*F*_(2,36)_ = 1.023, *P* = 0.370, *η*^2^ = 0.054), CONDITION (*F*_(1,18)_ = 0.456, *P* = 0.0508, *η*^2^ = 0.025), nor was there a significant SICF protocol × CONDITION interaction (*F*_(2,36)_ = 0.073, *P* = 0.930, *η*^2^ = 0.004). Significant differences existed between SICF 1.5 and SICF 4.5 (*P* = 0.020) post real tDCS, as well as between SICF 3.5 and SICF 4.5 (*P* = 0.046; Figure 4).

### Latency and Short Interval Intracortical Facilitation to Predict Responders to Transcranial Direct Current Stimulation

**Figure 5:**
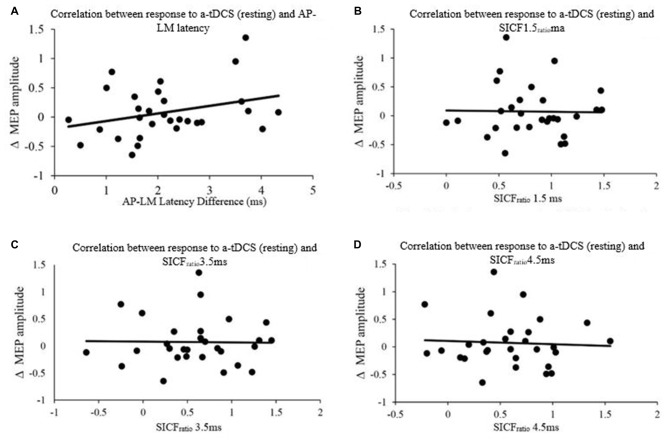
**Correlation between response to Anodal-tDCS (a-tDCS; resting) and (A) AP-LM latency, (B) SICF_ratio_ 1.5 ms, (C) SICF_ratio_ 3.5 ms and (D) SICF_ratio_ 4.5 ms**.

The latencies of the orientations (LM, AP and PA) as well as the latency differences between orientations (AP-LM, PA-LM and AP-PA) are displayed in Table 2. Pearson product moment correlations showed that response to a-tDCS in both active and resting muscle states was not related to latency difference (AP-PA, AP-LM, PA-LM) or SICF_ratio_ (1.5, 3.5. 4.5; all *P* > 0.05; Figure 5). Correlation data is displayed in Table 3.

**Table 2:** **Mean ± SE for latencies and latency differences of the latero-media (LM), anterior-posterior (AP) and posterior-anterior (PA) orientations, obtained at rest pre-tDCS (ms)**.

**LM (ms)**	**AP (ms)**	**PA (ms)**
15.91 ± 0.16	18.21 ± 0.19	16.78 ± 0.17
**AP-LM (ms)**	**PA-LM (ms)**	**AP-PA (ms)**
2.16 ± 0.19	0.72 ± 0.14	1.44 ± 0.18

**Table 3:** **Correlations between latency differences, SICF_ratio_, and response to a-tDCS**.

	AP-PA	AP-LM	PA-LM	SICF_ratio_ 1.5	SICF_ratio_ 3.5	SICF_ratio_ 4.5
**A-TDCS**	*r* = 0.098	*r* = 0.145	*r* = 0.102	*r* = 0.010	*r* = 0.057	*r* = 0.066
**(ACTIVE)**	*P* = 0.607	*P* = 0.446	*P* = 0.591	*P* = 0.958	*P* = 0.767	*P* = 0.736
**A-TDCS**	*r* = 0.166	*r* = 0.308	*r* = 0.261	*r* = −0.020	*r* = −0.016	*r* = −0.055
**(RESTING)**	*P* = 0.380	*P* = 0.098	*P* = 0.164	*P* = 0.916	*P* = 0.935	*P* = 0.775

## Discussion

We aimed to determine the individual variability of responses to a-tDCS using the difference in MEP latencies (in LM, PA and AP coil orientations), and compared it to paired-pulse TMS at I-wave periodicities. We hypothesized that both methods would correlate to the individuals’ response to a-tDCS. None of the MEP latency measures, nor any of the SICF measures correlated to the change in excitability following a-tDCS in resting or active conditions. This disagrees with our hypothesis that paired-pulse TMS at I-wave periodicity would be predictive of a-tDCS responses.

### Variability in a-tDCS Responses

Similar to Wiethoff et al. (2014), our results demonstrated that tDCS responses between individuals were highly variable, with approximately half the participants responding in a canonical manner to a-tDCS. The amount of I-wave facilitation and recruitment (Lang et al., 2011), N-methyl-D-aspartate (NMDA) receptor activity (Nitsche et al., 2003), as well as skull thickness and brain derived neurotrohpic factor (BDNF) polymorphisms (Antal et al., 2010; Opitz et al., 2015), could explain some of the variance of the tDCS after-effects. I-waves can be facilitated through a-tDCS if there is a sufficient amount of a-tDCS to induce intracortical changes which alters the after-effects of tDCS (Nitsche et al., 2005). If the amplitude of a-tDCS is insufficient, changes in NMDA receptor efficacy may not be induced (Nitsche et al., 2005). Differences in skull thickness, which increases resistance to tDCS, and BDNF polymorphisms, which make individuals more susceptible to externally induced plasticity, could both inhibit a-tDCS and therefore no after-effects would be induced (Nitsche et al., 2005; Antal et al., 2010; Opitz et al., 2015). This was observed in both the active and resting conditions (53% and 47% respectively), while a reduction in MEP amplitude occurred in approximately 20% of individuals for both conditions. Overall, our group average data did not show a facilitatory effect of a-tDCS on MEP amplitude which could have been attributed to the large variability in tDCS responses. This is not uncommon, with studies frequently reporting no facilitation in MEP amplitude, particularly when tDCS is applied in healthy populations (Horvath et al., 2015; McCambridge et al., 2015), and with no additional or concurrent motor stimuli (Hendy and Kidgell, 2014).

### Predicting a-tDCS Variability Based Upon I-wave Recruitment and Facilitation

**Figure 6:**
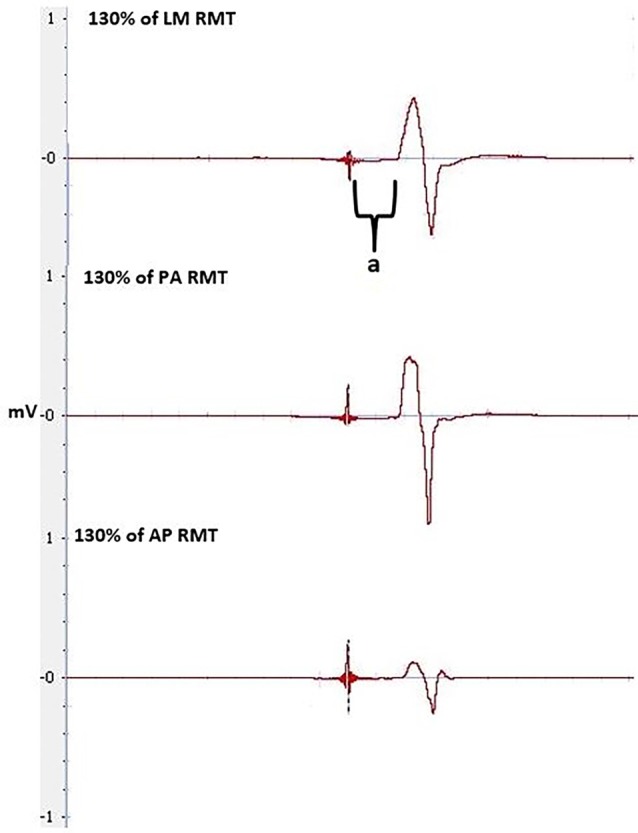
**MEPs at 130% of RMT at LM, PA and AP orientation, pre-tDCS, used to calculate latencies and latency difference.** Latency is between the stimulus to the onset of the MEP, indicated by **a**.

**Figure 7:**
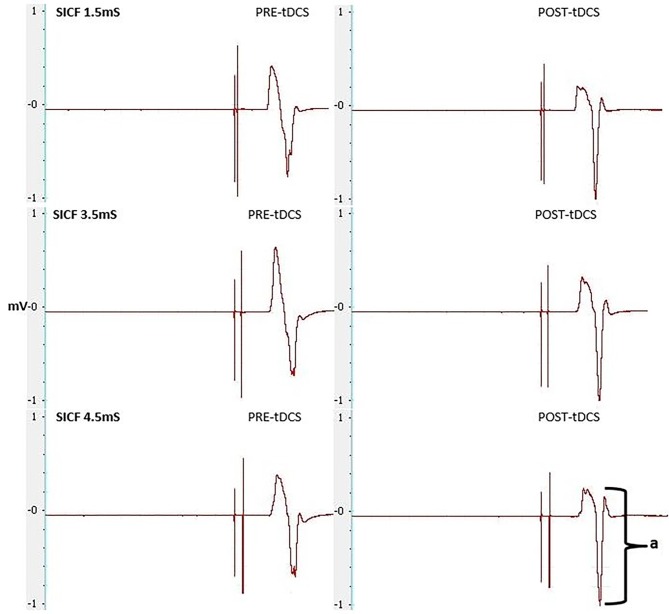
**MEPs elicited through SICF at 1.5, 3.5 and 4.5 ms from pre- and post-tDCS in the real condition. a** shows the amplitude of the MEP, calculated from peak to peak of the muscle response.

Our study did not show a correlation between AP-LM latency difference and response to a-tDCS. This is in contrast to the correlation reported by McCambridge et al. (2015). Methodological differences, such as determining MEP onset, defining the beginning and end of MEP latency (Figure 6), stimulation duration and electrode montage (dual vs. uni-hemisphere stimulation parameters) may, in part, explain some of the discrepancies between our results and previous studies (McCambridge et al., 2015). Our experiment was conducted in a comparatively large sample size of 30, using pre-programmed code based double blinding, as well as a previously established sham protocol to ensure scientific rigour (Gandiga et al., 2006). While the recruitment of I-waves may partially explain the variability of tDCS responses, we hypothesized that I-wave facilitation may also attribute to tDCS variability. Later I-waves are significantly facilitated for 4 min post a-tDCS, while I1-wave amplitudes are significantly facilitated for 2 min post a-tDCS (Lang et al., 2011). Later I-waves are thought to be generated from more superficial cortical layers than the I1 waves (Lang et al., 2011). Therefore, the cortico-cortical and thalamo-cortical fibers and interneurons may be activated more readily through a-tDCS. While the axons of these connections that produce the early I-waves are not activated as readily. This is because they are located deeper in the cortex. A possible explanation for this could be the dendritic model which states that early I-waves are activated through excitatory and inhibitory neurons in a pool, that synapse with pyramidal neurons in layer V (McCambridge et al., 2015).When sham tDCS was delivered, there was no relationship between the change in MEP amplitude at 130% of RMT and AMT in the PA orientation to the AP-LM latency difference, or to any of the SICF measures. This indicates that the changes in SICF measures in the real session (Figure 7) is likely to be an actual physiological effect, rather than measurement error or variability. Despite this, we did not find a correlation between the pre-tDCS SICF measure and the post-tDCS MEP response. Therefore, the present findings do not support the use of the SICF measures to determine tDCS variability.

**Figure 8:**
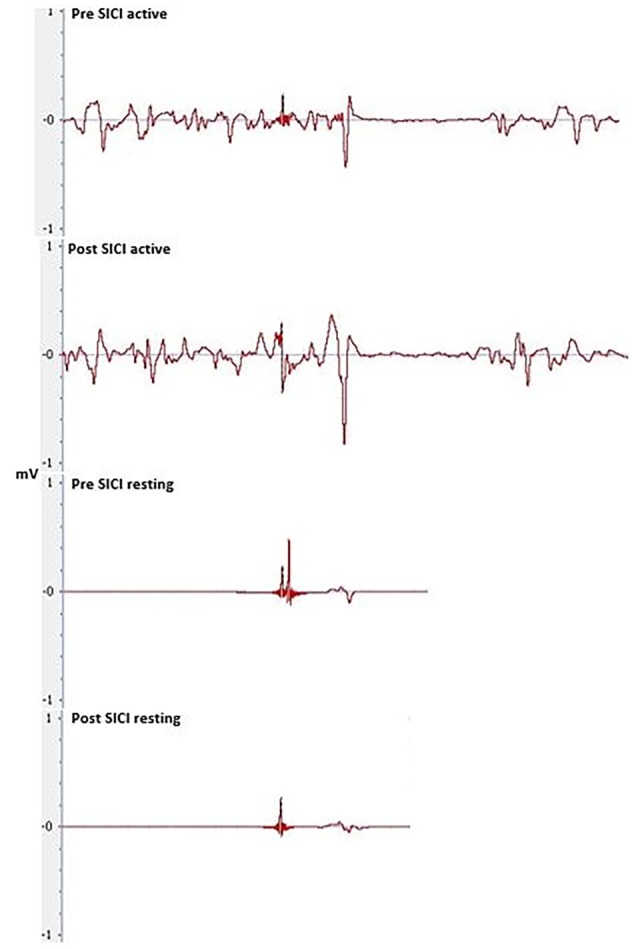
**Pre and post-tDCS MEPs elicited through SICI during resting and active conditions**.

**Figure 9:**
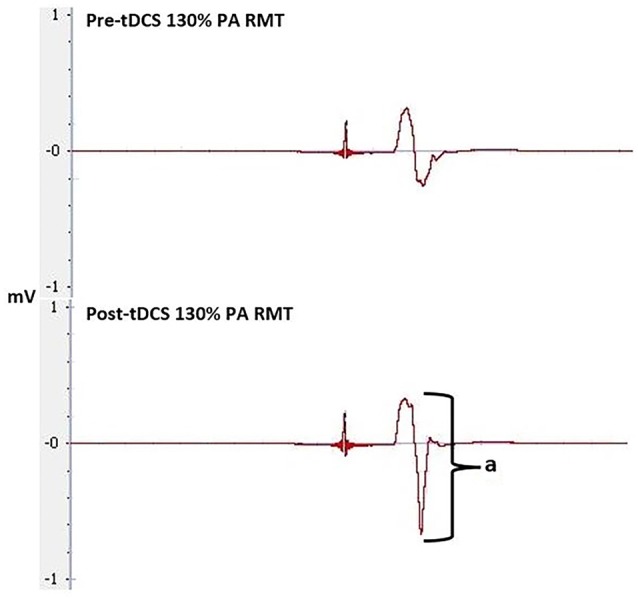
**Pre and post-tDCS MEPs elicited through 130% of RMT at the PA orientation.** Amplitude **(a)** was used to compare MEP size pre- and post-tDCS to determine tDCS effectiveness.

### Limitations and Conclusion

Several limitations in our study have to be acknowledged. While it is accepted that paired-pulse TMS at 1.5, 3.5 and 4.5 ms ISIs are able to target early and late I-waves, it is likely that these ISIs may not coincide with the optimal periodicities to elicit maximal facilitation of I-waves in every participant. It is suggested that a range of values (e.g., 1.3–1.7 ms, 3.0–3.5 ms and 4.3–4.7 ms) can also cause facilitation of I-waves that is individualized to each participant (Cash et al., 2009). Therefore, to accurately determine the relationship between I-wave facilitation and tDCS responses, individual I-wave response curves must be determined to elicit maximum I-wave responses. However, this may not be feasible to conduct on individual participants. Another potential limitation is the saturation or a ceiling effect of MEPs post-tDCS. It is possible that the %MSO intensity used for single-pulse TMS following tDCS may have resulted in maximal facilitation of MEPs. However this is unlikely in our study as the level of intensity used (130% AMT and RMT) has previously been used and intensities at which saturation occurs is typically between 170–190% of active or resting threshold (Ziemann et al., 1998; Goodwill et al., 2013; Pearce et al., 2013a,b). Using paired-pulse measures is another potential limitation as it has been shown these measures have an inherent level of inter-tester variability (Boroojerdi et al., 2000). All reasonable steps were taken by the researchers, who followed standard protocol (Rossini et al., 2015), to limit this variability when comparing to other literature. Finally, the amount of TMS pulses elicited, may account for some of the individual variability in responses to tDCS, as single-pulse TMS has been shown to have cumulative corticospinal excitability effects (Pellicciari et al., 2016).

In conclusion, our results showed that paired-pulse TMS at I-wave periodicity did not correlate to tDCS responses. Therefore paired-pulse TMS at I-wave periodicity may not be an appropriate measure to determine individual variability to tDCS responses. We demonstrated that the responses to 20 min of a-tDCS is highly variable, which is in line with previous studies. The latency difference in the AP-LM coil orientation and tDCS responses were also inconsistent with previous literature. This is likely due to methodological differences between studies. Overall, our findings highlight the need for more robust methods to determine tDCS responses in order to accurately predict if an individual may benefit from the application of tDCS.

## Author Contributions

W-PT and AMH conceptualized the idea for this study. NDN, W-PT and AMH collected the data and were all involved in the data analysis process. NDN, AMH, APR and W-PT were involved in the manuscript write up process.

## Conflict of Interest Statement

The authors declare that the research was conducted in the absence of any commercial or financial relationships that could be construed as a potential conflict of interest.

## Abbreviations

AMT: Active motor threshold
AP: Anterior-posterior
a-tDCS: Anodal-tDCS
BDNF: Brain derived neurotrohpic factor
CNS: Central nervous system
CS: Conditioning stimulus
c-tDCS: Cathodal-tDCS
D-waves: Direct waves
ECR: Extensor carpi radialis
EMG: Electromyography
fMRI: Functional magnetic resonance imaging
GABA_A_: Gamma-aminobutyric acid-A
ISI: Inter stimulus interval
I-waves: Indirect waves
LM: Latero-medial
M1: Primary motor cortex
MEP: Motor evoked potential
NMDA: N-methyl-D-aspartate
PA: Posterior-anterior
PET: Positron emission tomography
RMT: Resting motor threshold
sEMG: Surface electromyography
SICF: Short interval intracortical facilitation
SICI: Short interval intracortical inhibition
tDCS: Transcranial direct current stimulation
TES: Transcranial electrical stimulation
TMS: Transcranial magnetic stimulation
TS: Testing stimulus.
