# Supplementary material for: Measures to Predict The Individual Variability of Corticospinal Responses Following Transcranial Direct Current Stimulation
Source: Front Hum Neurosci. 2016 Oct 6;10:487. doi: 10.3389/fnhum.2016.00487 (PMC5052268; doi:10.3389/fnhum.2016.00487)
Supplement: Supplementary file 1 [file DataSheet_1.docx]

Indicate on the scales below the level of sensation/pain/tingling you felt during stimulation.

**Pain**

**2**

**6**

**0**

**No Pain**

**3**

**4**

**7**

**8**

**9**

**1**

**10**

**Worst Possible Pain**

**5**

**Moderate Pain**

**Tingling**

**5**

**Moderate Tingling**

**0**

**No Tingling**

**2**

**6**

**3**

**4**

**7**

**8**

**9**

**1**

**10**

**Worst Possible Tingling**

**Itching Sensation**

**0**

**No Itching Sensation**

**5**

**Moderate Itching Sensation**

**2**

**6**

**3**

**4**

**7**

**8**

**9**

**1**

**10**

**Worst Possible Itching Sensation**

**Burning Sensation**

**10**

**Worst Possible Burning Sensation**

**5**

**Moderate Burning Sensation**

**0**

**No Burning Sensation**

**2**

**6**

**3**

**4**

**7**

**8**

**9**

**1**

**What stimulation do you believe you received? Circle one.**

**Real tDCS Sham tDCS**
